# Parallel Transmission of Distributed Sensor Based on SCTP and TCP for Heterogeneous Wireless Networks in IoT †

**DOI:** 10.3390/s19092005

**Published:** 2019-04-29

**Authors:** Weifeng Sun, Shumiao Yu, Yuanxun Xing, Zhenquan Qin

**Affiliations:** School of Software, Dalian University of Technology, Dalian 116620, China; wfsun@dlut.edu.cn (W.S.); yusm1995@outlook.com (S.Y.); xingyuanxun2017@163.com (Y.X.)

**Keywords:** IoT, parallel transmission, packet error rate, path switching, wireless sensor network

## Abstract

Sensors in the Internet of Things (IoT) generate large amounts of data, which requires high-speed data transmission. In order to achieve the parallel transmissions of the wireless sensor network on the transmission layer, the performance of stream control transmission protocol (SCTP) and transmission control protocol (TCP) in the wireless sensor network under different packet error rates was simulated and compared. A dynamic multipath handover method for SCTP (MS-SCTP) was proposed to improve the transmission performance, which selects the transmission path according to the packet error rate and the retransmission ratio in the sender’s buffer. The TCP and SCTP protocol switching method (TCP-SCTP) was proposed to detect the current network traffic and adjust the MS-SCTP or TCP method. Analysis and simulation results show that MS-SCTP and TCP-SCTP could improve network throughput and reduce packet loss rate. MS-SCTP and TCP-SCTP can be combined with other technologies and channel allocation algorithms to improve network traffic.

## 1. Introduction

The Internet of Things (IoT) [1] is not only an important part of the new generation of information technology but also an important stage of development in the "information" era. Sensor technology is a key technology in the IoT. A great deal of real-time data generated by sensors need to be scheduled [2] and securely transmitted [3] to the sink nodes through wireless or wired networks. Distributed sensors are deployed in different areas, and the data are harvested in distributed sink nodes. These data need to be transmitted to one or more certain destinations through heterogeneous networks. The data transmissions among the wireless and wired networks face the high latency, high packet loss rate and other problems. In IoT, it is important to improve transmission efficiency and reduce packet loss for these distributed data. The quality of information transmission between sensors is critical, and high-quality data transmission among sensors or sink nodes are necessary in wireless sensor networks (WSN) [4] and IoT. CyberGIS [5] deals with location based data on the IoT, which could solve the location based services. The sensors’ data harvested from different areas are stored in different locations. The Fog computing [6] can solve the sensing data on edge networks to enhance the data transmission efficiencies. Software defined network (SDN) based sensor network [7] can also balance the data transfer congestions in the IoT environments by the centralized scheduling. In these scenarios, the techniques of methods all require high speed and high performance wired or wireless data transmissions. IoTs and the fifth generation (5G) [8] visions aim at providing massive connectivity, high access speed, low latency, and quality of service (QoS). Various types of networks and incompatible networks also bring challenges to the transmission of heterogeneous networks [9]. In order to improve the transmission speed of wireless networks, parallel transmission technologies on different layers are proposed. For instance, Multiple-input multiple-output (MIMO) and channel assignment algorithms [10] on the media access control address (MAC) layer; multi-path routing algorithms [11] on the network layer; SCTP and multipath TCP (MTCP) [12] on the transport layer; data separation methods on the application or the higher layer; and cross layer [13,14] designs for multipath transmission. In addition, network coding methods [15,16] are used to leverage and balance the data transmission traffic. Sundararajan J K et al. [17] propose a new mechanism called the TCP/NC that incorporates network coding into TCP with only minor changes to the protocol stack, thereby allowing incremental deployment. At the same time, the connection establishment process of SCTP is more complicated.

In [18], we proposed a dynamic multipath switching method for SCTP to improve the efficiency of information transmission in heterogeneous networks and reduce packet loss rate. The packet loss rate of each transmission link is used to predict the transmission quality of each link and dynamically adjust the main link in advance to improve the efficiency of information transmission. In this paper, we propose a new scheme that could improve the transmission quality by the switch of TCP and SCTP. Compared to our previous work, we compared the average throughput, real-time throughput, and stability between TCP and SCTP in detail. This work proposes the combined TCP-SCTP and also proposes the path selection methods based on the sender’s buffer. MTCP [19] is used in multimedia and collaborative computing [20]. SCTP has the multi-host and multi-flow features. The multi-host feature can fully use the network link resources and improve the transmission efficiency. Multi-flow features can process multiple request information parallel to control information, and therefore data can also be separated and allocated to different streams. To some extent, it improves the response efficiency and the transmission efficiency and improves the utilization rate of network resources. SCTP supports multiple transmission modes and is suitable for multi-path parallel transmission [21], and the performance of SCTP is better than TCP in the 4G network [22]. However, TCP and SCTP have their own disadvantages. SCTP improves TCP’s congestion control strategy. In parallel transport, SCTP adopts conservative congestion control, which cannot match the SCTP multi-path transmission. In the wireless network environment and the wired-cum-wireless network environment, because of SCTP’s conservative congestion control mechanism and detection mechanism, TCP tends to be more stable. After the establishment of the coupling of SCTP, though the secondary path remains idle, only the primary path can be used for data transmission. C Ramiro et al. [23] proposes two practical parallel approaches of the subspace marginalization with interference suppression detector for large MIMO systems. Qin K et al. [24] proposes a flow splitting algorithm, which splits a data flow to multiple sub-flows by extending the open-flow protocol in SDN. A multiple-path routing algorithm is also proposed to implement the multi-path parallel transmission in the paper. Wegner M et al. [25] present SCTPCL, a convergence layer protocol for the bundle protocol, which brings reliability and low-power to delay tolerant networks (DTN) implementations.

In this work, we simulated TCP and SCTP with different packet error rate (PERs) in a heterogeneous network and compared the performance of TCP and SCTP with different network quality. Based on the analysis and simulation results, the multipath switching SCTP scheme (MS-SCTP) and the TCP-SCTP method were proposed to improve the network transmission quality. Our contributions in this work can be summarized as follows:

- Based on the analysis and simulation results of the IEEE 802.11 wireless multi-hop network, the performance and problems of SCTP and TCP under different network environments are analyzed and compared.
- A multipath switching scheme MS-SCTP is proposed to apply SCTP to data transmission in high speed networks. MS-SCTP judges the network transmission situation of each link based on packet loss rate, and then selects the main line in advance to ensure the link transmission quality.
- TCP and SCTP are more suitable for wireless cable network transmission. Based on packet loss rate, the TCP-SCTP method predicts the current link transmission condition. When the link transmission quality becomes worse, the TCP-SCTP switch process improves the transmission quality.
- Simulation and analysis of TCP-SCTP and MS-SCTP in network transmission show that TCP-SCTP and MS-SCTP improve throughput and stability, and improve network transmission quality.

The remainder of the article is organized as follows. Section 2 shows the simulation results of TCP and SCTP in different PER scenarios, which are displayed with the performance of SCTP and TCP in the wireless-cum-wired network. Section 3 details the MS-SCTP and the TCP-SCTP methods. Section 4 determines the analysis and simulation. Section 5 concludes this article and presents the future work.

## 2. Problem Statement

The wireless sensor networks and the IoT located in distributed sink nodes or data servers generate data. The distributed data in different locations require high-speed, high-quality wireless or wired transmission. To state this problem, a sink node (data server) is abstracted as an end node in the network, and the heterogonous network path is abstracted as a link. The end node is a server that has the stable energy supply and can deal with some computing problems. The links could be wired links or wireless links. There are enormous wireless links, such as Zigbee, 5G, Wi-Fi, and so on, while there is bit error rate in the wireless transmissions. In this paper, we selected IEEE 802.11 serious standers (IEEE 802.11 g/n/ac) as the wireless links. We aimed at transmitting data from some end nodes to other end nodes through unreliable wireless links efficiently.

Parallel transmission is one of the options for increasing wireless transmission rates. SCTP was proposed to push the limitations of TCP. It inherits many of the advantages of TCP and adds some features that TCP does not possess. In wireless multi-hop networks, transmission performances at different PERs are different. In order to demonstrate the performance of transmission over unreliable wireless links, simulations of different parameter settings were performed on TCP and SCTP.

### 2.1. Simulation Scenarios

There is bit error rate (BER) in wireless transmissions, and packet error rate (PER) occurs as long as BER exists. In order to demonstrate the transmission performance of SCTP and TCP over different links on wireless wired networks and wireless multi-hop networks, simulations were performed using NS2.35.

**Figure 1:**
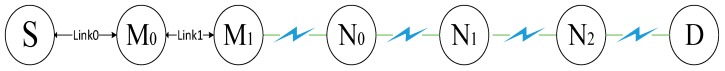
Simulation scene topology.

The simulations were done on the IEEE 802.11 based on the wireless network, and the wireless network was connected to the wired network. The simulation topology was a link with seven nodes. In Figure 1, from left to right, the start node, node M_0_, and node M_1_ were connected by the wired network; node M_1_ also had a wireless interface, which connected the end node (on the right side) through wireless node N_0_, node N_1_, and node N_2_. In the simulations, the start node and the end node ran TCP protocol and SCTP protocol separately, and file transfer protocol (FTP) was used to send data on the application layer. During the simulations, the error-module was used to simulate the PER in the paths. Table 1 shows the detail parameter configurations. The PER was between 0% and 50%. The simulation results show that with the change of PER value in the wireless network, the packet delay, throughput, and packet loss rate in the network had certain changes.

### 2.2. Comparison of the Average Throughput

**Figure 2:**
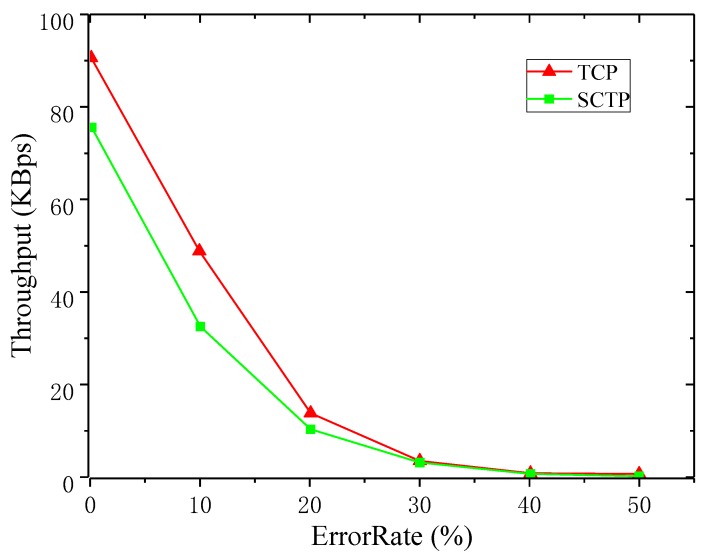
Comparison of average throughput between SCTP and TCP.

When analyzing the average throughput of TCP and SCTP, the PERs changed from 0% to 50%. Figure 2 shows the comparison between the average throughput of SCTP and TCP at different PERs.

When PER increased, the corresponding average throughput of SCTP and TCP decreased rapidly, but the average throughput of SCTP was always greater than that of TCP. When the packet error rate reached 30%, the average throughput of both SCTP and TCP dropped below 10 KBps. Due to the poor link condition, neither SCTP nor TCP were ideal in data transmission rate. At this time, the average throughputs of both were relatively low. By comparing the real-time throughput with the average throughput, SCTP could make better use of network bandwidth and transmit data better with the same PER.

### 2.3. Comparison of the Real-Time Throughput

**Figure 3:**
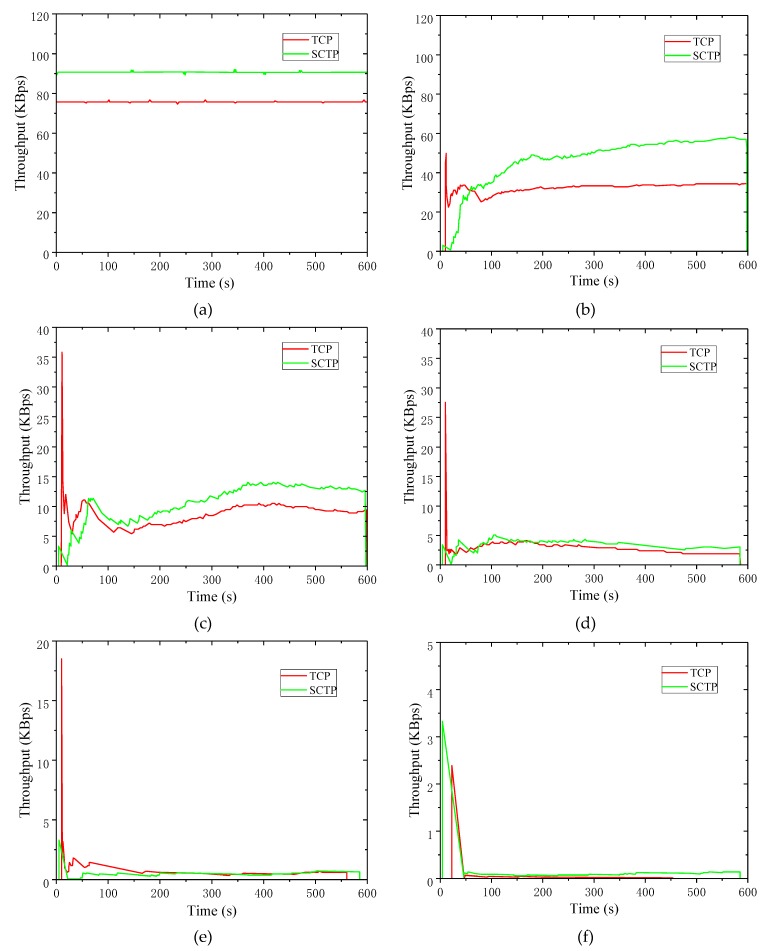
Real-time throughput comparison when the error rate is 0–50%. (**a**) Packet error rate (PER): 0%; (**b**) PER: 10%; (**c**) PER: 20%; (**d**) PER: 30%; (**e**) PER: 40%; (**f**) PER: 50%.

Figure 3 shows the real-time throughput comparison of SCTP and TCP under different PERs in networks that combine wire-wireless and multi-hop situations. The PERs were selected as 0%, 10%, 20%, 30%, 40%, and 50%, respectively.

Figure 3a shows when the PER was zero, the throughputs of SCTP and TCP were 90 KBps and 77 KBps separately during the 600 s simulation time. The real-time throughput of SCTP was greater than TCP. In Figure 3f, when the PER was 50%, the throughputs of TCP and SCTP were both less than 4 KBps, and the throughput of the SCTP was always higher than TCP during the simulation time. When the PER was 10%, the average throughput of SCTP was 50 KBps, while the average throughput of TCP was 30 KBps in Figure 3c. On the whole, the real-time throughput of SCTP was higher than TCP. However, with the increment of PER, the advantages of SCTP became smaller. In Figure 3e, when the PER reached 40%, the throughputs of SCTP and TCP were close to zero. When the data PER was higher than 10%, the real-time throughput of TCP showed a significant trend of increasing at the beginning of data transmission in Figure 3. Moreover, the real-time throughput of TCP was much larger than SCTP, which could be explained by the connection mechanism of TCP and SCTP. SCTP required four handshakes to establish a coupling when establishing a connection. However, TCP required only three handshakes to establish a connection. The connection process of SCTP was more complex and took more time than TCP, therefore TCP had a higher throughput than SCTP when data started to transmit.

### 2.4. Comparison of the Packet Loss Rate

**Figure 4:**
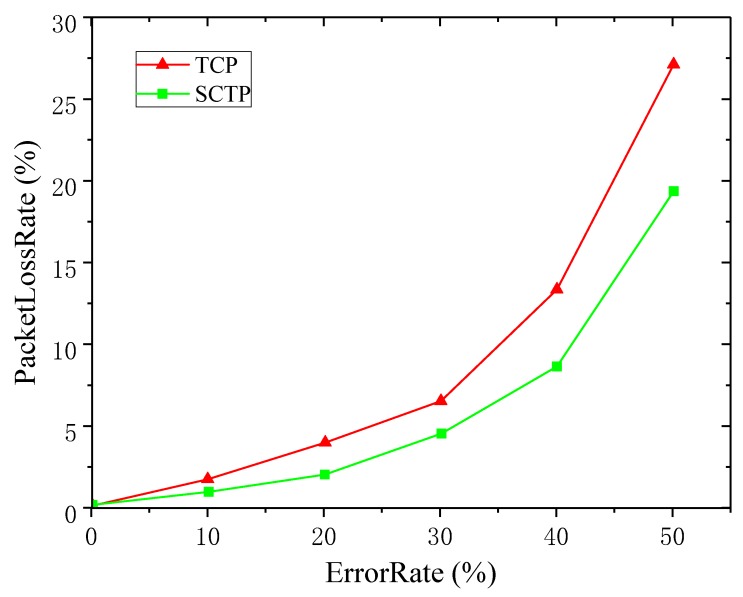
Comparison of the packet loss rate between SCTP and TCP.

Figure 4 shows the packet loss rates of SCTP and TCP under different PERs. By analyzing the trace file, the number of packets lost and the total number of packets sent could be counted to calculate the packet loss rate.

In the case of different packet error rates, with the increase of PER, the probability of packet loss of TCP and SCTP increased. However, the packet loss rate of TCP was higher than SCTP at the same PER. In order to ensure the reliability of the data, both SCTP and TCP needed to retransmit the lost packet data. Therefore, the resource consumption of the retransmission packet of SCTP was lower. With the increase of PER, the trend of TCP packet loss rate increased much more rapidly than SCTP. The retransmission mechanism is a way to ensure data reliability. In the process of packet retransmission, the retransmission mechanism increased the burden of the link. Low packet loss rate is helpful to improve data transmission speed. Therefore, using SCTP for data transmission could effectively improve the transmission efficiency. It also showed that SCTP had unique advantages over TCP.

### 2.5. Comparison of the Stability

**Figure 5:**
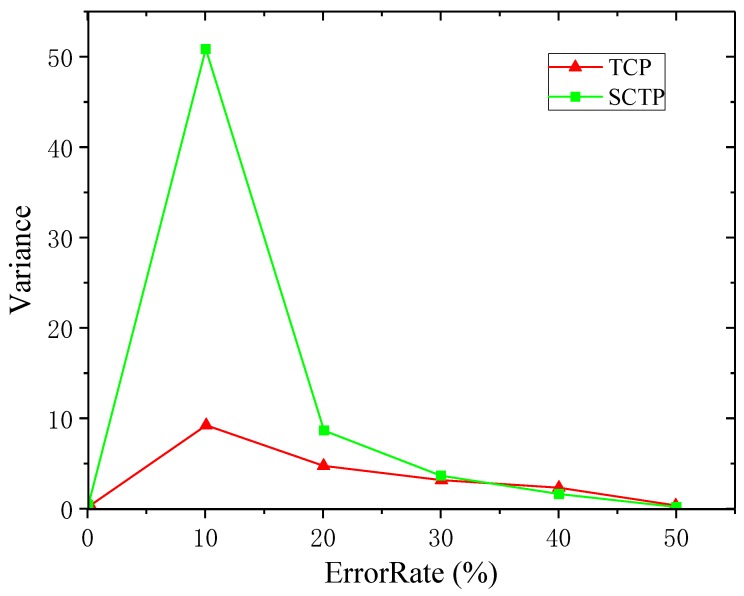
Compare the throughput variance between SCTP and TCP.

The variance of throughputs of SCTP and TCP under different PERs was calculated to describe the throughput fluctuations of SCTP and TCP. A total of six simulations were performed. Figure 5 shows the comparison of throughput variance between SCTP and TCP under different PERs.

Figure 5 shows that the SCTP throughput variance was greater than TCP in most cases as the PER changed. In the interval of data PER between 0% and 10%, the throughput variance of SCTP and TCP was on the rise. As the data PER increased, the network situation became worse. At this time, the throughput fluctuation was larger when data transmission was conducted. The data PER was between 10% and 50%, and the throughput variance curves of SCTP and TCP were both in the decline stage. When the PER reached 10%, the real-time throughput of both decreased significantly. While Figure 5 shows that the real-time throughput curve fluctuated less, the variance of real-time throughput decreased, and the results were consistent.

The analysis above shows that, under the condition of the same PER, SCTP in the process of data transmission had larger real-time throughput and average throughput. However, from Figure 5, the SCTP throughput variance curve was basically above the TCP, which meant that TCP provided more stable and reliable data transmission than SCTP. Due to the fact that SCTP adopted a conservative congestion control scheme based on TCP, SCTP multipath transmission and multi-flow characteristics did not match with SCTP’s congestion control strategy. Although SCTP provided the maximum throughput relative to TCP, TCP was more widely used in practical applications when considering the stability of data transmission. In this paper, a combined TCP and SCTP multi-path switching scheme TCP-SCTP was proposed to improve the throughput and stability of data transmission.

## 3. Algorithm Design and Analysis

### 3.1. MS-SCTP Method

SCTP adopted a conservative congestion control strategy and could not fully exploit link redundancy. The TCP-SCTP method did the link selection according to a pre-judgment of the current network transmission state. When the current network condition was about to enter the congestion state, the MS-SCTP method was called to perform link selection before the main line completely failed. This greatly improved the efficiency and reduced the burden on each path. The following is a detailed analysis and algorithm design of MS-SCTP.

#### 3.1.1. Model Description

**Figure 6:**
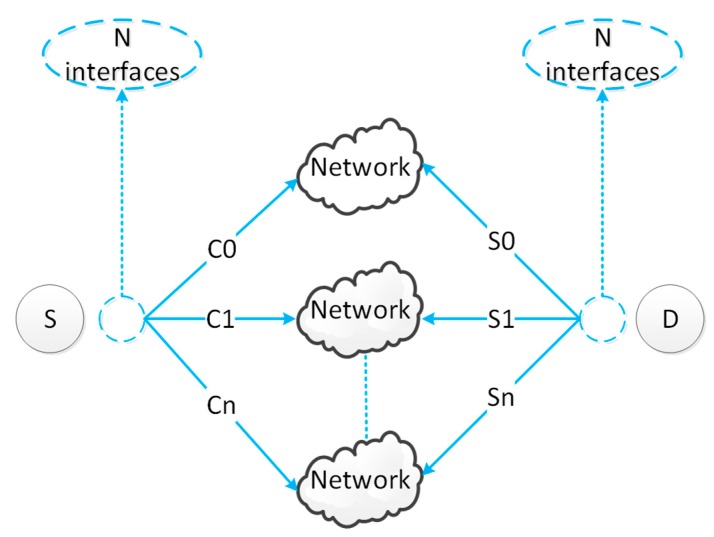
SCTP multi-path transmission model.

SCTP could establish multiple paths between two communication nodes, each of which could be used for data transmission. The SCTP multi-path transmission model is shown in Figure 6. After the SCTP was established, multiple communication paths were established between the two nodes, and data could be transmitted on multiple streams. Multi-path communication required multiple interfaces, which could be heterogeneous network interfaces. During the transmission, if an error occurred in the stream, the data in other streams could be transmitted normally. In addition, erroneous data could be retransmitted in the same stream or in other streams. The multi-objective optimization problem selected a candidate path. SCTP managed these multiple streams in a unified manner. In the process of data transmission and reception, in order to ensure the order of data, each data block in SCTP was equipped with a sequence number. The sequence number was maintained by a stream sequence number (SSN) and a transport sequence number (TSN). Data loss and data sequence in the SCTP scheme were detected through TSN and SSN.

#### 3.1.2. Parameter Descriptions in MS-SCTP Method

The MS-SCTP method selected a primary path according to the packet loss rate. In the method, some relevant parameters are shown in Table 2.

MS-SCTP defined array Last to store the packet loss rate on the latest path information of each path, and it also defined a Max array to store the largest packet loss rate information of each path. The Last array and Max array element initialization was zero. CurrentNumber recorded the current number of the main path. PathNumber recorded selected primary path numbers. CurrentNumber and PathNumber initialized to zero. In the process of establishing a connection, the first primary path was the path that selected zero.

#### 3.1.3. Algorithm Design

The MS-SCTP method determined the primary path through the packet loss rate recorded last time in each path and the maximum packet loss rate in the link. It is shown in Algorithm 1.

- Line 1 indicates that the switching algorithm began when the main path needed reselection.
- Line 2 indicates that the link probe needed to be performed before switching between the master and the slave paths. If the path was found unreachable, the Max array and the last array values corresponding to the path were updated. When the path was not reachable, the value of the Max array and the corresponding position in the Last array was set to 101.
- Lines 3 and 4 show that the algorithm traversed the Max and the Last arrays and recorded the Max and Last values for each path.
- Line 5 indicates that the path with the smallest result was selected by calculating 0.25 × Max + 0.75 × Last.
- Lines 6 through 14 show that if more than one alternative path was selected, the algorithm needed to confirm whether the alternative paths package included the current main path. If the alternate paths included the current primary path, it was not necessary to re-switch the primary path. If the alternative paths did not include the current main path, the scheme randomly selected one as the new main path and recorded it.
- Lines 15 through 18 indicate that when making a primary path switch, the algorithm first determined whether the recorded PathNumber variable had the same value as the current primary PathNumber. If so, then it did not have to switch. If not, the algorithm set the new primary path based on the value of the recorded path number and updated the CurrentNumber value.

**Algorithm 1. MS-SCTP Method**
**Input:** Max[], Last[], CurrentNumber **Output:** CurrentNumber 1:   Method begin 2:    Detect the reachability of the path, update the Max array and Last array 3:    Search the Max array and Last array 4:    Record the max value as Max and record the last value as Last 5:    Calculate 0.25 * Max + 0.75 * Last and select the paths with the smallest value 6:    If results have more than one path number 7:       If results contains the CurrentNumber 8:         Let PathNumber = CurrentNumber 9:         Else Choose one at random and record the PathNumber 10:          End Else 11:         End If 12:         Else Record the PathNumber 13:         End Else 14:    End If 15:    If CurrentNumber == PathNumber 16:         No longer switch 17:         Else Set the primary path according the PathNumber 18:              Let CurrentNumber = PathNumber 19:         End Else 20:    End If 21:   End Method

### 3.2. TCP-SCTP Switch Process

The third part compared the performance indicators of SCTP and TCP in the process of data transmission. Although SCTP had a higher throughput than TCP, TCP was superior to SCTP in terms of stability. Meanwhile, TCP was faster than SCTP connection during connection establishment. We proposed the TCP-SCTP algorithm, which alternated TCP and SCTP protocols in data transmission. The TCP-SCTP method judged performance of the protocol undergoing data transmission and decided whether to switch to another protocol for data transmission. The TCP-SCTP switch process could switch the current protocol to another protocol. The following is a detailed analysis and process design of TCP-SCTP switch process.

#### 3.2.1. Parameter Descriptions in TCP-SCTP Switch Process

The TCP-SCTP switch process selected protocol according to the current protocol. In the scheme, some relevant parameters are shown in Table 3.

The TCP-SCTP switch process distinguished what was the currently used protocol type. ProtocolType variable recorded the type of the current protocol. Using the SCTP protocol, the ProtocolType value was one. When using the TCP protocol, the ProtocolType value was zero. The program initialization used SCTP to transmit data, and the ProtocolType initialization was one.

#### 3.2.2. Process Design

The TCP-SCTP switch process was used to select protocol and store the type of the current protocol. It is shown in Algorithm 2.


**Algorithm 2. TCP-SCTP Switch Process**
**Input:** ProtocolType **Output:** ProtocolType 1:   Process begin 2:      If ProtocolType == 0 3:            Program apply SCTP to establish the connection 4:            Let ProtocolType = 1 5:            Else 6:            Program apply TCP to establish the connection 7:            Let ProtocolType = 0 8:            End Else 9:      End If 10:   End Process

- Line 1 indicates that the process started running when the TCP-SCTP method judged that it needed to make protocol switch.
- Line 2 shows that the process needed to judge which type of protocol was running. If the current ProtocolType value was zero, it indicated that the TCP protocol was currently running. The protocol needed to be switched to SCTP.
- Line 3 indicates that the current transport protocol was set to SCTP.
- Line 4 was used to record the protocol type for process switching. The current usage protocol was set as SCTP, and the ProtocolType was assigned a value of one. The TCP-SCTP switch process ended.
- Line 5 indicates that Line 2 was false. The current ProtocolType value was one, therefore the SCTP protocol was currently running. The protocol needed to be switched to TCP.
- Line 6 shows that the current transport protocol was set to TCP.
- Line 7 was used to record the protocol type for process switching. The current usage protocol was set as TCP, and the ProtocolType was assigned a value of zero. The TCP-SCTP switch process ended.

### 3.3. TCP-SCTP Method

The TCP-SCTP method was used to judge the current network transmission quality. If the network was about to be blocked, the MS-SCTP or the TCP-SCTP switch process was called to improve the network state. This method measured network transmission by packet loss rate. The detailed analysis and the algorithm design of TCP-SCTP method are as follows.

#### 3.3.1. TCP-SCTP Method Description

From the analysis results of the third part, after SCTP and TCP PER reached 30%, their real-time throughput and average throughput dropped to under 5 KBps, especially after the arrival of the PER rose to 40%. This suggests that the link status became very poor. As shown in Figure 4, when the PER reached 30%, the packet loss rate of TCP and SCTP was around 5%. In this algorithm, 5% packet loss rate was selected as the critical point of path switching.

The TCP-SCTP method first needed to obtain the packet loss rate of packets, which was the standard procedure to judge the network link status. With the trace file format, the method could calculate the packet loss rate by counting the number of lost packets and the number of sent packets. After the packet loss rate reached 5% in the link, the throughput of packets in the link decreased greatly, and the packet loss events increased. The current protocols did not effectively improve network congestion. The TCP-SCTP method could improve the current network transmission by calling the MS-SCTP method or the TCP-SCTP switch process and continuing to observe the transmission quality of the link.

From Figure 3, when the packet loss rate was less than 5%, the PER was less than 30%. There was still a throughput of more than 10 KBps in the link. At this point, it could wait for a period of time to detect the packet loss rate in the link. If the packet loss rate continued to increase, it indicated the link was becoming worse. If the packet loss rate increased three times, the MS-SCTP method or the TCP-SCTP switch process was called by the TCP-SCTP method. The detection cycle was set by the DetectSlot parameter.

If the packet loss rate detected was greater than 5%, or the packet loss rate continued to increase, it indicated that the current situation of the network was poor. It needed to decide as soon as possible whether the network link was in better or worse condition in order to take appropriate measures. For this reason, a DetectSlot parameter was introduced on the interval of detecting packet loss rate. DetectSlot was used to represent the frequency at which the TCP-SCTP method calculated the packet loss rate. The method adjusted the interval time of packet loss rate dynamically according to the network status. If the network was normal, the time interval for the next packet loss detection was set longer. If the network was poor, the next detection should have been done as soon as possible. Each change in DetectSlot was a multiple of two.

If the packet loss rate detected reached 5%, the next time interval was set to half the time interval for this time (DetectSlot/2). If the packet loss rate detected was less than 5%, but the packet loss rate detected this time was larger than the packet loss rate detected last time, then the time interval of the next detection should also have been set to half of the time interval of this detection (DetectSlot/2). If the packet loss rate detected this time was lower than the packet loss rate detected last time, which indicated that the network condition started to improve, then the next detection time was twice as long as the interval of this detection (DetectSlot × 2).

#### 3.3.2. Parameter Descriptions in TCP-SCTP Method

The TCP-SCTP method predicted the network conditions according to the packet loss rate. In the method, some relevant parameters are shown in Table 4.

In the TCP-SCTP method, the DetectSlot variable recorded the calculation interval of packet loss rate, initially set to 16 s. Each change in the DetectSlot was a multiple of two. The packet (segment) loss rate could be calculated by the sender. The Count variable recorded the consecutive increase times of packet loss rate. The LastRate and the Rate recorded the previous packet loss rate and the current packet loss rate. The Max array was used to store the maximum packet loss rate information on each path. The array subscript corresponded to the path label, and if the path was unreachable, it was set to 101 (which was corresponding to the Max array). The TCP-SCTP method defined the Min array to store the minimum packet loss rate information on each path. The elements in the Min array and in the Max array were initialized to zero.

#### 3.3.3. Algorithm Design

The TCP-SCTP method was used to predict the network transmission quality, and the MS-SCTP method or the TCP-SCTP switch process was called to improve the network transmission. It was an information record and an algorithm initial process. It is shown in Algorithm 3.


**Algorithm 3. TCP-SCTP Method**
**Input:** trace file (computing packet loss rate), DetectSlot **Output:** Rate 1:   Method begin 2:    Wait DetectSlot time 3:    Record the current packet loss rate in Rate, update LastRate be Rate 4:    While true 5:      Wait DetectSlot time, then record the current packet loss rate in Rate 6:      If Rate > 5% 7:         We need to apply MS-SCTP method or TCP-SCTP switch process 8:         Let LastRate = Rate 9:         Let Count = 0 10:          Let DetectSlot = DetectSlot 11:          Continue 12:      End If 13:      Else If Rate >= lastRate 14:             Let LastRate = Rate 15:             Let Count = Count + 1 16:             Let DetectSlot = DetectSlot / 2 17:             Else 18:               Let LastRate = Rate 19:               Let Count = 0 20:               Let DetectSlot = DetectSlot * 2 21:             End Else 22:          End If 23:          If Count >= 3 24:            We need to apply MS-SCTP method or TCP-SCTP switch process 25:            Let Count = 0 26:          End If 27:      End Else 28:    End While 29:    Procedure run 600 s, and then end procedure 30:   End Method

- Line 1 indicates that the algorithm started executing after the initial connection was established.
- Line 2 indicates that the method waited for the default detection interval time before starting to detect network conditions.
- Line 3 indicates that after the algorithm calculated the packet loss rate, it recorded the packet loss rate into the Rate variable, and at the same time updated the value of LastRate so that it could compare with the next time detection of the packet loss rate so as to predict the state of the network.
- The algorithm needed to repeatedly detect packet loss rate of packets, thus Line 4 kept the algorithm running by setting the loop condition to true.
- After the algorithm started running, it initially got a packet loss rate value, and Line 5 waited for the DetectSlot time before detecting the network status.
- The algorithm got the packet loss rate again after the time interval of the DetectSlot. After the calculation packet loss rate, Line 6 started to judge the packet loss rate. If the packet loss rate was greater than 5%, then the network situation was poor.
- Line 7 shows that the algorithm called the previously defined MS-SCTP method or the TCP-SCTP switch process. After calling the MS-SCTP method or the TCP-SCTP switch process, we needed to update some parameter variables.
- Line 8 was used to update the LastRate value. It detected the new packet loss rate of the loop every time, thus it needed to update the LastRate every time as well.
- Line 9 shows this algorithm set Count value to zero. Because the Count was used to record the times, the PER continued to rise. After switch another protocol, the Count is set to zero.
- Line 10 set the interval between the next detection of packet loss rate. The data transmission improved after switching the protocol. Therefore, the DetectSlot kept the current value.
- Line 11 indicates that the algorithm should have continued the next loop after switching the protocol instead of continuing to perform the following judgment.
- Line 13 represents the algorithm’s processing scheme if the detected packet loss rate was less than 5%. Firstly, the packet loss rate obtained at present was compared with the packet loss rate recorded last time to predict network transmission quality. If the packet loss rate of the current packet was greater than the packet loss rate recorded last time, the network condition was worse than that of the last time.
- Line 14 was used to update the LastRate value for comparison with the packet loss rate next time.
- Line 15 was used to update the value of Count, and the algorithm needed to add one to the value of Count.
- Line 16 updated the value of DetectSlot. The network was getting worse, thus it set the value of DetectSlot to half the original value.
- Line 17 indicates that the current packet loss rate was less than the last recorded packet loss rate.
- The same Line 18 was used to update the LastRate value.
- Line 19 was used to update the value of Count. The packet loss rate did not keep increasing, thus the algorithm needed to reset the value of Count to zero.
- Line 20 was used to update the value of DetectSlot. The algorithm determined that the network condition was getting better, thus it did not need to frequently detect the trend of the network condition. It set the value of DetectSlot to twice the original value.
- After obtaining the new packet loss rate every time, Line 23 started to judge whether or not the packet loss rate kept increasing for three times through the value of Count. If the packet loss rate kept increasing for three times, it was considered that the network condition kept getting worse.
- Line 24 shows the algorithm calling the MS-SCTP method or the TCP-SCTP switch process.
- Line 25 indicates that the Count value was reset to zero after the protocol switch.
- Line 29 shows that after the simulation process lasted 600 s, the program execution process ended.

The MS-SCTP method also needed to open up two arrays spaces of size *n* to store the maximum packet loss rate and the latest packet loss rate corresponding to each path, respectively. In the process of choosing a path, if there were multiple alternative paths, it needed an array with the maximum length of n to store the information of multiple alternative paths, and the space complexity was O(*n*).

In the process of choosing the path, the algorithm needed to choose the path with lower packet loss rate. Therefore, it needed to compare the data in the array. In the process of comparison, the algorithm adopted the algorithm of quick sorting. When *n* connections were made, *n* pieces of data needed to be compared in the array, and the average time complexity of the quicksort algorithm was O(*n*lg2*n*). At this point, in the process of algorithm execution, the time spent on switching paths was the main time consumption, and the other time required could be regarded as *t*. Therefore, the time required for each switch was *t* + *n*lg2*n*, thus the time complexity of the algorithm was O(*n*lg2*n*).

## 4. Simulations and Analysis

### 4.1. Simulation Results of MS-SCTP Method

**Figure 7:**
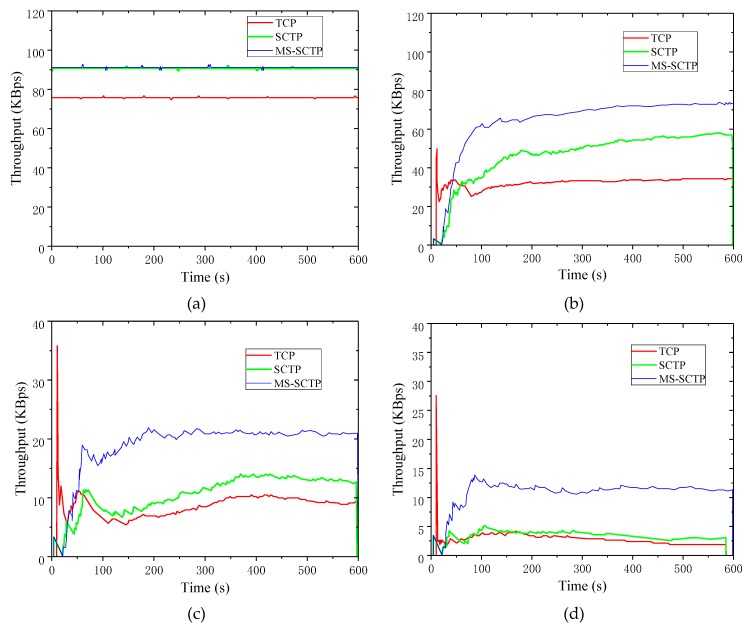

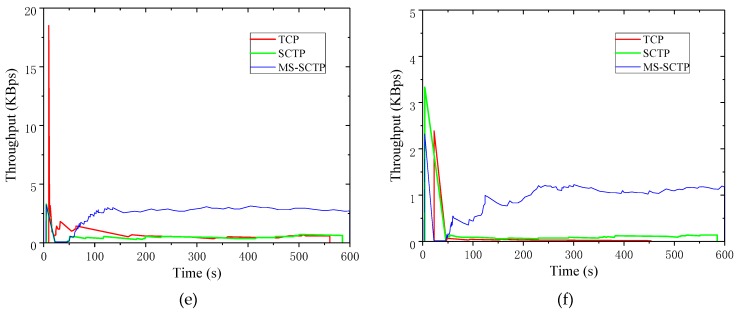
Real-time throughput comparison when the error rate was 0–50%. (**a**) PER: 0%; (**b**) PER: 10%; (**c**) PER: 20%; (**d**) PER: 30%; (**e**) PER: 40%; (**f**) PER: 50%.

In simulations, the same simulation scenario was used as 3.1, and the PERs were 0%, 10%, 20%, 30%, 40%, and 50%. The metrics throughput and packet loss rates of TCP, SCTP, and MS-SCTP were used in the simulations to analyze the advantages of MS-SCTP. Figure 7 shows the real-time throughput of the three schemes when the PER changed from 0% to 50%.

As shown in Figure 7a, when the PER was zero, the real-time throughput curve of the SCTP multi-path switching scheme was basically the same as that of SCTP alone. The throughput difference between them was small. When the PER was zero, the external influence on data transmission was little. When transferring data, it was affected by a large number of packet errors. The condition that triggered the MS-SCTP did not occur. SCTP allowed the primary path chosen at the time of the initial coupling to be used to transfer data all the time. As a result, throughput remained stable. Since no path switching was involved, the default choice of a constant main path was used to transfer data. As the PER increased, such as in Figure 7b, the MS-SCTP showed advantages. The PER was greater than 10%. Different from the TCP-SCTP switch process, the MS-SCTP method switched the primary path to improve the network condition instead of adjusting the current network congestion by switching another protocol. After the path switch, MS-SCTP found a path that was better than the current path. Figure 7c displays that when the PER reached 20%, the MS-SCTP method could keep the throughput stable around 20 KBps, while the throughputs of the TCP protocol and the SCTP native protocol were only about half of this scheme. From Figure 7d, the throughputs of the TCP protocol and the SCTP native protocol were significantly reduced or even close to zero. However, the throughput of the MS-SCTP scheme was only slightly lower than the PER when the PER was 20%, and it still stayed around 10 KBps stably, not declining rapidly due to the deterioration of the link condition. When the PER went up to 40% in Figure 7e, the real-time throughput curve under the MS-SCTP scheme still existed. However, at this point, the throughput under the TCP and the native SCTP protocols had begun to approach zero. Even when the PER was as high as 50%, as shown in Figure 7f, the real-time throughput of MS-SCTP was also 1 KBps.

**Figure 8:**
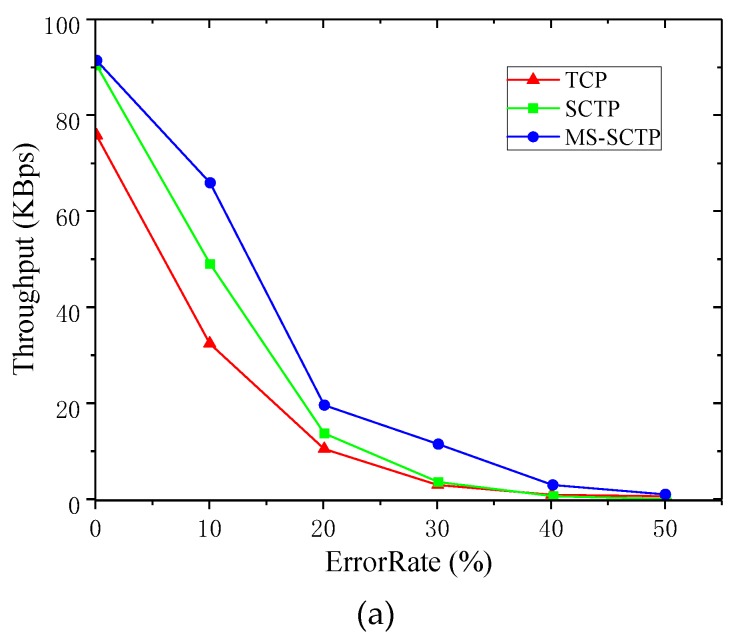

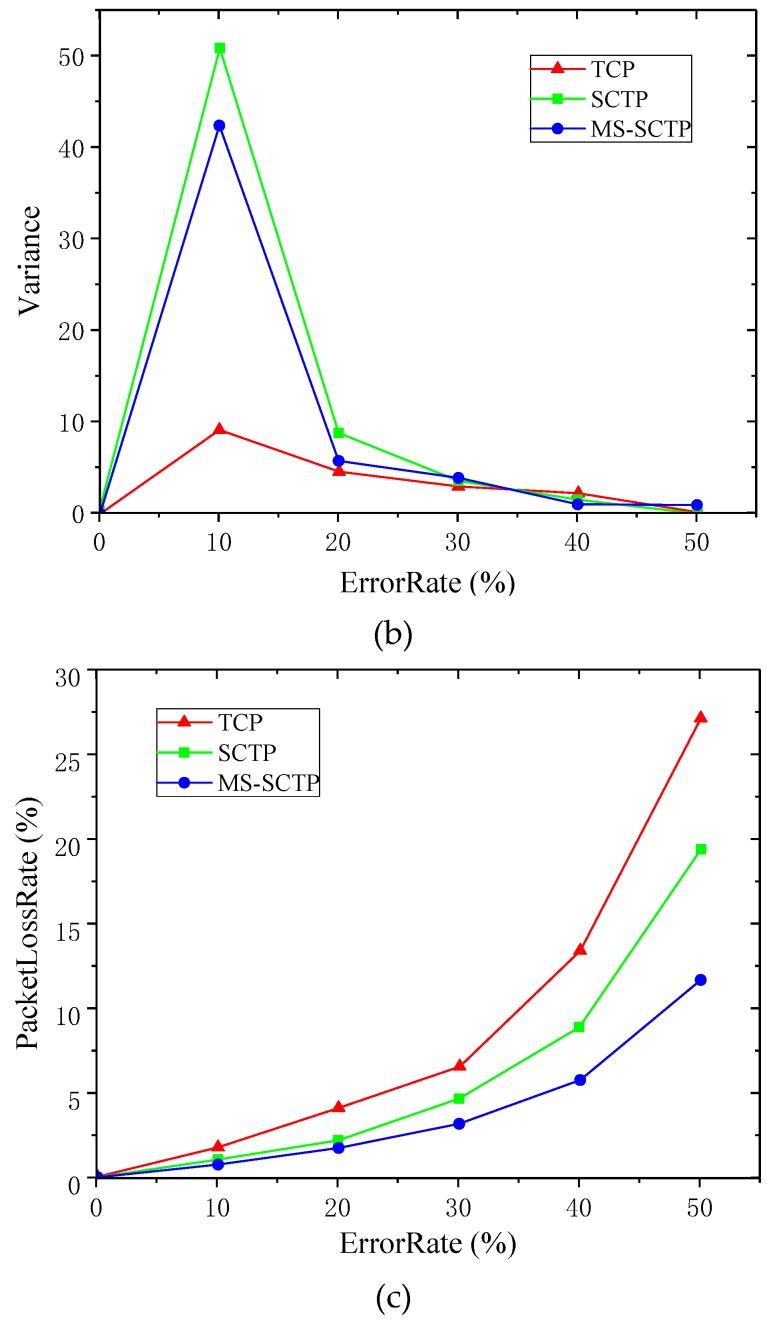
Comparison of three schemes’ performances. (**a**) Average throughput; (**b**) throughput variance; (**c**) packet loss rate.

Figure 8 shows the comparison of other metrics among the three schemes.

Figure 8a shows the comparison of average throughput with the three schemes at different PERs. At the PER of 0%, the throughput curve looked the same when using the MS-SCTP schemes as it did when using the SCTP native protocols for data transfer. However, the average throughput of the SCTP multipath switching protocol was slightly higher. As the PER increased, the average throughput of the three schemes decreased rapidly. However, from the range of 0% to 10%, the decline trend of average throughput was much slower when the MS-SCTP scheme was used. This shows that the scheme played an important role in the improvement of data transmission. When the PER reached 30%, the average throughput of the MS-SCTP scheme still had obvious advantages. After the PER reached 50%, the condition of each link became very bad, and the average throughput approached zero.

From Figure 8b, when using the MS-SCTP scheme for data transmission, the throughput variance variation trend was similar to that when using SCTP. The scheme was an improvement on the SCTP protocol, and it still retained some features of SCTP. SCTP was more volatile in throughput changes than TCP and remained a feature of MS-SCTP. The MS-SCTP had a smaller variance in throughput than the SCTP native protocol, but it was larger than TCP. All three curves showed an upward trend before the PER reached 10%. When the PER reached 20%, the throughput fluctuation range of the MS-SCTP was close to TCP. At this point, the throughputs of the TCP and the native SCTP protocols were greatly reduced. However, the MS-SCTP also had the advantage of relatively large throughput and relative stability while maintaining a large throughput, which was the advantage of this scheme. When the PER reached 40%, the throughput variance curve of MS-SCTP was at the bottom, and its throughput was still the highest. The MS-SCTP was indeed a great improvement over SCTP, which not only improved throughput but also ensured better stability to some extent.

As shown in Figure 8c, the packet loss rate of the three schemes had the same trend under different PERs, which all increased with the rise of PER. However, in each case of the same packet loss rate, the packet loss rate of the MS-SCTP scheme was lower than that when using the TCP and the SCTP native protocols. This shows that the TCP-SCTP played a certain role in the process of predicting the merits and the demerits of the network, and that the prediction of the scheme had certain accuracy and improved the reliability of data transmission. For the MS-SCTP scheme, it was inevitable that the increase of PER would cause more packets to be lost in the link. With the increase of PER, the MS-SCTP did not have the sharp increase of packet loss rate as the SCTP and the TCP protocols did, but the change was gentler. This also shows that it could better avoid congestion in the link by predicting the network status.

### 4.2. Simulation Results of TCP-SCTP Switch Process

In simulations, the same simulation scenario was used as 3.1, and the PERs were 0%, 10%, 20%, 30%, 40%, and 50%. The metrics throughput and packet loss rates of the TCP, the SCTP, and the TCP-SCTP switch process were used in the simulations to analyze the advantages of the TCP-SCTP switch process.

**Figure 9:**
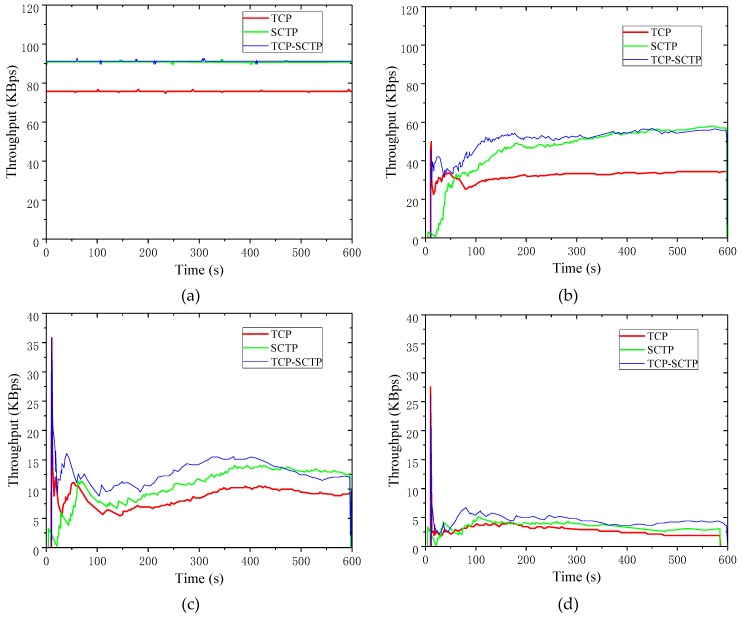

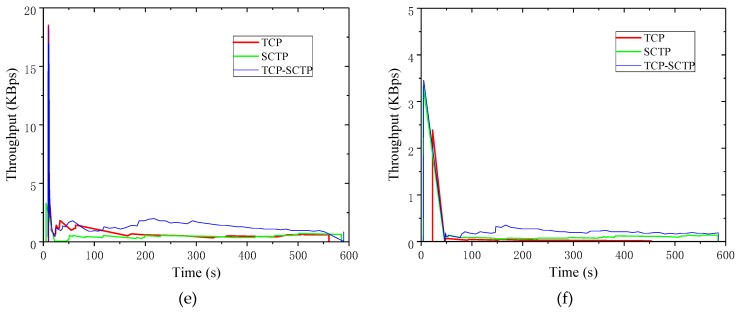
Real-time throughput compare when the error rate was 0–50%. (**a**) PER: 0%; (**b**) PER: 10%; (**c**) PER: 20%; (**d**) PER: 30%; (**e**) PER: 40%; (**f**) PER: 50%.

Figure 9 shows the real-time throughput of the three schemes when the PER changed from 0% to 50%. As shown in the figures, the throughputs of the three schemes were occasionally similar (as in Figure 9a), while the throughput was different most of the time.

From Figure 9a, when the data PER was zero, the TCP-SCTP switch process was used for data transmission, and its real-time throughput curve basically coincided with the real-time throughput curve when SCTP was used alone for data transmission. The real-time throughput could be assumed to be the same, except for a few time points where there was a large difference in real-time throughput. This was related to the scheme initialization settings using the SCTP protocol for data transfer. In Figure 9b, when the PER was more than 10%, the TCP-SCTP switch process had certain advantages in throughput compared to using SCTP and TCP alone. The throughput of the TCP-SCTP switch process reached 60 KBps; the throughput of SCTP was 50 KBps. From Figure 9c, where the PER was greater than 20%, the TCP-SCTP switch process was not obvious in terms of performance improvement. All three curves went up and down around 10 KBps. From Figure 9d, when the PER reached 30%, the throughputs of TCP and SCTP and the TCP-SCTP switch process were under 10 KBps. When the data PER reached 40% in Figure 9e, the real-time throughput of the scheme was also close to zero.

**Figure 10:**
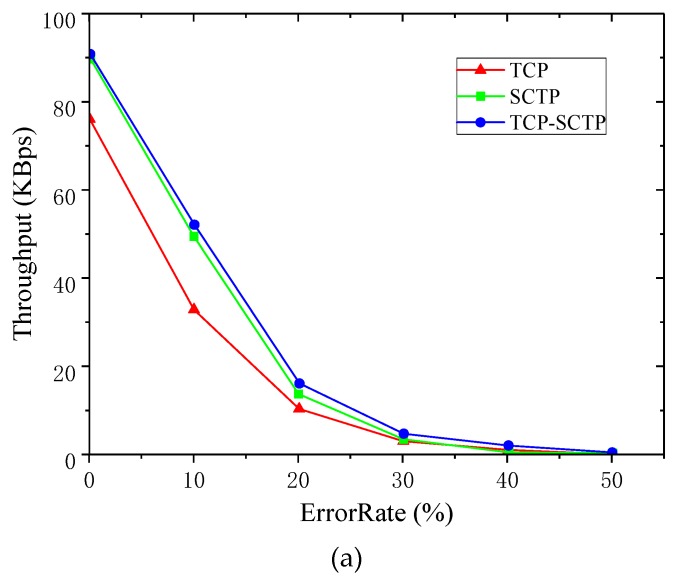

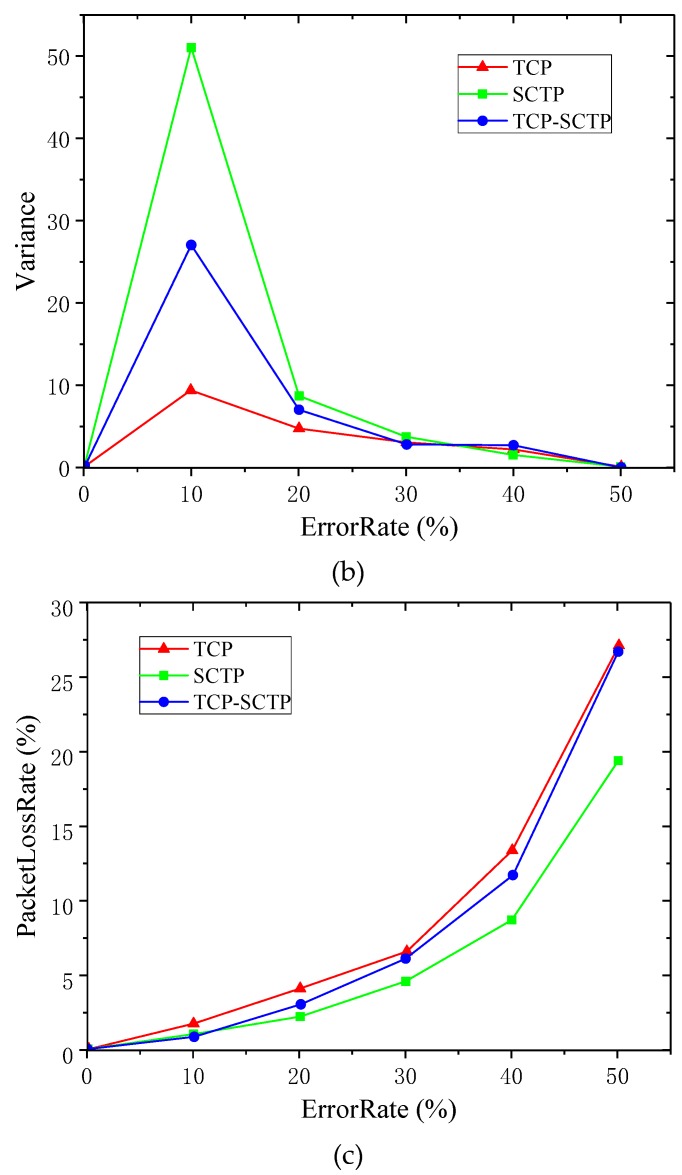
Comparison of three schemes performance. (**a**) Average throughput; (**b**) throughput variance; (**c**) packet loss rate.

Figure 10 shows the comparisons of other metrics among the three schemes.

Figure 10a shows the comparison results of the average throughput of the three schemes under different data PERs. When the data PERs increased, the average throughput rapidly decreased, either using the TCP-SCTP switch process or using them alone for data transmission. The average throughput of the TCP-SCTP switch process was always higher than that of TCP and SCTP alone for data transmission. When the data PER was more than 30%, the average throughput of all three schemes dropped below 10 KBps. When the data PER reached 40%, the data transmission performance was very poor.

Figure 10b shows the comparison of throughput variance under different PERs. The throughput variance for data transfer using the TCP-SCTP switch process was larger than TCP but smaller than SCTP. The throughput fluctuation of the TCP-SCTP switch process was between the curves of transmission using SCTP and TCP. When the PER was at 30%, the variance of the throughput of the scheme was slightly less than that of TCP. All three curves had similar trends. Before the PER reached 10%, the throughput variances of all three were on the rise. The data PER was between 10% and 50%, and the throughput variance curves of all three were in the decline stage.

Figure 10c shows the packet loss rate of three schemes under different PERs. In the case of different PERs, with the increase of data PERs, the probability of data packet loss in the three schemes increased. However, in each case of the same packet loss rate, the packet loss rate of TCP was higher than that of SCTP, and the loss rate curve of the TCP-SCTP switch process was in the middle. The packet loss rate of the TCP-SCTP switch process was small when the PER was small. The packet loss rate of the TCP-SCTP switch process was the lowest when the PER was less than 10%. With the continuous increase of the PER, the packet loss rate of the TCP-SCTP switch process increased faster and faster. With the increase of PER, the number of packets lost in the link increased, which easily triggered the condition of protocol switching between SCTP and TCP and also resulted in poor performance of the scheme.

### 4.3. Implementation Analysis

This study simulated the MS-SCTP and the TCP-SCTP switch process proposed in this paper through network simulator version 2 (NS2). The TCP-SCTP method used packet loss rates to predict network conditions. In NS2, the packet loss rate was calculated by analyzing the trace file, which was used as a reference standard for packet retransmission analysis. In a practical application, when the transmission time out or packet loss, the sender retransmits the lost packet. In this paper, by calculating the proportion of retransmitted packets, the proportion of packets lost during data transmission was known. In this way, the TCP-SCTP method could do the statistics of retransmission packets more conveniently. The simulation took packet retransmission rate as a condition of protocol or path switching.

From the analysis above, the MS-SCTP scheme had a significant effect on the throughput improvement under the same network conditions, regardless of the real-time throughput or the average throughput. At the same time, according to the throughput variance curve, although this scheme did not show satisfying stability when PER was relatively low, this scheme showed advantages in stability with higher packet loss rate. In terms of packet loss rate, it also optimized the SCTP protocol. The MS-SCTP scheme was the best of all three schemes. For the more conservative congestion control strategy of SCTP, the MS-SCTP improved the efficiency of congestion control to a certain extent, thus the multi-host feature of SCTP could play a better role. At the same time, the SCTP link redundancy features played a greater role. When SCTP was coupled, it was not necessary to wait for the primary path to fail completely before considering reselecting a primary path from the path. This not only increased the utilization of each path but also reduced the burden on each path.

The TCP-SCTP method predicted the network status through packet loss rate. The current protocol was not ideal for congestion control, thus the TCP-SCTP switch process was carried out. However, the scheme did not directly change the status of the path itself or find a better path. Instead, it hoped to adjust the congestion of the current network by switching to another protocol so as to achieve more efficient data transmission. However, the scheme itself did not guarantee that network congestion could be dealt with by switching the protocol. Therefore, the optimal effect achieved by each switch of the protocol did not exceed the optimal effect achieved by the protocol alone for the adjustment of network conditions. From the analysis above, in the same network conditions, the switching scheme between SCTP and TCP could improve the throughput, either in real time or in average time. The TCP-SCTP switch process showed slightly better performance. At the same time, from the throughput variance curve, the TCP-SCTP switch process facilitated the transmission of data when the throughput performance was more stable. In terms of packet loss rate, it also performed better than TCP alone for data transmission. Therefore, we can consider using the TCP-SCTP switch process to improve system throughput. It is more stable than using the SCTP protocol alone for data transmission, and it also has a greater throughput than using the TCP protocol alone for data transmission.

## 5. Conclusions and Future Work

Data generated by sensors in the Internet of Things and geographic information system (GIS) need to be transmitted over heterogeneous wireless wired networks. The sensor data collected by the wireless multi-hop network can be sent in parallel to increase the data transmission rate. In multi-hop wireless networks and hybrid networks, it is necessary to choose a reliable and efficient transport layer protocol for data transmission. It not only increases the data transfer rate but also increases the throughput of the system. When network congestion occurs, the current network protocol is changed to improve the network transmission status. The performance comparison of SCTP and TCP in a wireless wired network is simulated. In most cases, SCTP can achieve higher throughput and reliability than TCP. However, the transmission stability of SCTP is worse than TCP, and it takes a longer time to build the connection than TCP. This paper proposed the TCP-SCTP method to solve the defects of the two protocols. When the current network state became poor, the current protocol was converted to adjust the network transmission state. The TCP-SCTP switch process improved TCP throughput and SCTP stability. At the same time, the MS-SCTP method for SCTP multi-path optimization was proposed. MS-SCTP improved the multi-path conversion in the previous SCTP protocol. By calculating the packet loss rate in each network path, the state of each path network was predicted, and the optimal path was selected. This greatly reduced the latency and packet loss due to the congestion of the main path. MS-SCTP showed better performance in throughput, packet loss, and stability in data transmission. The TCP-SCTP method predicted the state of each network path by calculating the packet loss rate of each network path. The MS-SCTP or the TCP-SCTP switch processes were called by the TCP-SCTP method when the network was in poor condition.

In future studies, we will consider how to improve the performance of the TCP-SCTP method and how to use the SCTP multipath for data transmission at the same time. After selecting the main path, we will assist the data transmission from all paths at the same time. The TCP and the SCTP multipath switching methods will be combined for simulation tests to verify their performance in improving data transmission performance. The MIMO and application slicing methods will be considered to accelerate the data transmission speed with MS-SCTP.

## Figures and Tables

**Table 1:** Parameters Settings.

Parameter	Value
Wireless protocol	IEEE 802.11
Time slot	20 μs
Wireless bandwidth	1 MBps
Wired bandwidth	5 MBps
Link delay	2 ms
SCTP maximum transmission unit (MTU)	1500 B
SCTP chunk size	1468 B
Simulation time	600 s
Error rate type	Packet error rate
Rate values	0%~50%

**Table 2:** Parameter descriptions of the MS-SCTP method.

Parameter	Description
Max []	Record the maximum packet loss rate for each path
Last []	Record the latest packet loss rate for each path
CurrentNumber	Record the current primary path number
PathNumber	Record the selected new primary path number

**Table 3:** Parameter settings of the TCP-SCTP switch process.

Parameter	Description
ProtocolType	Record the protocol type

**Table 4:** Parameter settings of the TCP-SCTP method.

Parameter	Description
DetectSlot	Record the detection interval of packet loss rate
Count	Record the consecutive increase times of packet loss rate
LastRate	Record the previous packet loss rate
Rate	Record the current packet loss rate
Max[]	Record the maximum packet loss rate for each path
Min[]	Record the minimum packet loss rate for each path
